# Place des gestes associés à l’ostéotomie de Scarf dans le traitement de l’hallux valgus

**DOI:** 10.11604/pamj.2018.31.148.15993

**Published:** 2018-10-29

**Authors:** Zied Bellaaj, Skander Ben Dhia, Mohamed Allagui, Issam Aloui, Youssef Othmen, Makram Zrig, Mustapha Koubaa, Abderrazek Abid

**Affiliations:** 1 Service d’Orthopédie-Traumatologie, CHU Fattouma Bourguiba Monastir, Faculté de Médecine de Monastir, Tunisie

**Keywords:** Avant-pied, hallux valgus, ostéotomie de Scarf, ostéotomie phalangienne, ostéotomie Weil, Forefoot, hallux valgus, scarf osteotomy, phalangeal osteotomy, weil osteotomy

## Abstract

Devant la multiplicité des techniques proposées pour le traitement de l'hallux valgus, nous proposons d'évaluer l'ostéotomie Scarf associée ou non à une ostéotomie phalangienne et/ou une ostéotomie de Weil. Il s'agit d'une étude rétrospective de 29 patients, dont un cas bilatéral, opérés entre 2011 et 2016 par une ostéotomie de Scarf du premier rayon avec un geste associé dans 80% des cas. Les résultats ont été analysés selon la satisfaction des patients, l'indice de Groulier et les mesures radiologiques. Le score global de Groulier donnait une bonne évaluation objective du résultat prenant en compte les données radiologiques et anatomiques qui influencent le résultat final en cas d'insuffisance de correction. Le recul moyen était de 3 ans et 5 moins. Une diminution significative du valgus phalangien (de 34,17% à 16,1%), du métatarsus varus (de 15,13% à 9,93%) et de l'angle articulaire distal métatarsien (de 17,63% à 12,73%) étaient retrouvés. Les patients sont satisfaits et très satisfaits dans 83% des cas. Les complications sont dominées par l'hypo-correction dans 13,3% des cas et nous n'avons noté aucun cas de pseudarthrose ou de nécrose de la tête de M1. Nos résultats sont comparables à ceux de la littérature. Nous insistons sur le caractère surtout fonctionnel de la chirurgie de l'hallux valgus qu'il convient d'intégrer dans une correction globale de l'avant-pied. L'ostéotomie de Scarf nécessite une technique rigoureuse, elle est fiable par ses résultats et présente comme limite les grosses déformations surtout de l'angle articulaire distal métatarsien.

## Abstract (en)

In view of the multiplicity of the proposed techniques for hallux valgus correction, we recommend to evaluate scarf osteotomy associated or not with phalangeal osteotomy and/or Weil osteotomy. We conducted a retrospective study of 29 patients, including a bilateral case, undergoing scarf osteotomy of the first ray with associated treatments in 80% of cases between 2011 and 2016. Results were analyzed on the basis of patients’ satisfaction, Groulier index and radiological measurements. The overall Groulier score showed good objective assessment of the end results based on radiological and anatomical data influencing the end results in case of insufficient correction. The mean follow-up period was 3 years and 5 months. A significant reduction in phalangeal valgus (from 34.17% to 16.1%), in metatarsus varus (from 15.13% to 9.93%) and the distal metatarsal joint angle (17.63% to 12.73%) were obtained. Patients were satisfied and very satisfied in 83% of cases. Complications were dominated by hypocorrection in 13.3% of cases and no case of pseudarthrosis or of M1 head necrosis was reported. Our results are comparable to those reported in the literature. We particularly insist on the functional role of hallux valgus surgery that should be included in overall forefoot deformity correction. Scarf osteotomy requires rigorous technique. It gives reliable results, with limitations related to major deformities, especially of the distal metatarsal joint angle.

## Introduction

L'hallux valgus est une pathologie fréquente qui peut être responsable d'une gêne fonctionnelle et esthétique. Le diagnostic est facile, basé sur la clinique et confirmé par la radiologie. La chirurgie de l'hallux valgus doit s'intégrer dans le cadre d'une prise en charge globale de l'avant-pied. L'objectif de notre travail est d'analyser nos résultats, de soulever les différents facteurs influençant ces résultats et de montrer l'intérêt des gestes associés à l'ostéotomie Scarf.

## Méthodes

Nous avons mené une étude rétrospective intéressant les patients opérés pour hallux valgus symptomatique entre 2011 et 2016 tout en excluant le pied rhumatoïde. Dans ce travail, 29 patients ont été retenus dont un patient opéré des deux côtés, soit 30 pieds opérés au total. Les évaluations pré-opératoires et post-opératoires, ont été recueillies par le même examinateur, et comportaient un volet clinique en décharge puis en charge et un volet radiologique. L'évaluation radiologique était basée sur la méthode de Miller en estimant sur les clichés de face l'angle métatarso-phalangien (M1-P1), le métatarsus varus (M1-M2), l'étalement de la palette métatarsienne (M1-M5), l'angle articulaire métatarsien distal (DMMA), le valgus interphalangien (P1-P2), l'index métatarsien de Viladot [1], la position des sésamoïdes selon la stadification de Seite [2] et la congruence articulaire métatarso-phalangien en fonction des critères de Pigott [3]. Les radiographies de profil en charge ont permis de calculer l'angle de l'arche interne de Dijan Annonier [4] et l'angle de Schnepp [5] pour chaque rayon. La technique chirurgicale était la même pour tous les malades et comportait un premier temps de libération externe du complexe métatarso-phalangien, puis une exostosectomie, une ostéotomie de Scarf de M1 avec une dérotation fixée par deux vis dorso-plantaires et enfin une résection osseuse et une capsulorraphie avec recentrage des sésamoïdes. Les gestes associés étaient une ostéotomie phalangienne du premier rayon métaphysaire basale de Diebold [6] ou diaphysaire synthésées par des agrafes à mémoire de forme et/ou une ostéotomie cervico-capitale Weil [7] des rayons externes fixée par vis. L'appui est donné dès le lendemain dans des chaussures de Barouk et l'appui total est autorisé à partir de la troisième semaine. L'appréciation radio-clinique et fonctionnelle au dernier recul minimal de 12 mois est basée sur les critères de Groulier [8]. Les tests statistiques ont été réalisés à l'aide du logiciel SPPSS version 20.0.

## Résultats

Notre série comporte 26 femmes et 3 hommes avec un âge moyen de 36 ans au moment de la chirurgie. Le motif de consultation était des troubles essentiellement fonctionnels représentés par les douleurs et la gêne au chaussage. Des métatarsalgies ont été signalés dans 67% des cas. L'examen clinique pré-opératoire a objectivé que 53% de nos patients avaient un avant-pied de morphotype égyptien avec une bursite du gros orteil dans la quasi-totalité des cas. Les sésamoïdes n'étaient pas centrés dans tous les cas. Des signes d'arthrose au niveau de la métatarso-phalangienne du gros orteil étaient présents seulement dans 13% des cas. Une ostéotomie de P1 a été associée dans 50% des cas alors que l'ostéotomie cervico-capitale de Weil a été pratiquée dans 60% des cas. Au dernier recul moyen de 41 mois, les métatarsalgies ont disparu dans 50 % des cas et seulement réduites dans 40% des cas. L'association d'une ostéotomie cervico-capitale était significativement corrélée à la persistance ou la disparition de ces métatarsalgies. Le nombre d'avant-pieds de morphotype égyptien a diminué au profit du type carré qui a passé de 20% à 46,7%. En fait, l'ostéotomie phalangienne nous a permis de modifier significativement le morphotype de l'avant-pied. Sur le plan radiologique, une correction significative des différentes mesures angulaires est retrouvée comme le montre le Tableau 1. Une réaxation des sésamoïdes a été constatée dans la majorité des cas. On a noté la présence d'une corrélation entre la disparition des métatarsalgies et le score global de Groulier. De même ce score était corrélé à la correction de l'angle métatarso-phalangien. L'ensemble des complications observées dans notre série est résumé dans le Tableau 2. Des troubles de corrections ont été notés dans 20% des cas. A noter l'absence d'ostéonécrose de la tête de M1 et de pseudarthrose de l'ostéotomie qu'elle soit métatarsienne ou phalangienne.

**Tableau 1:** évolution des mesures angulaires après la chirurgie

		M1-P1	M1-M2	M1–M5	DMMA	P1-P2
Pré-opératoire	Moyenne	34,17°	15,13°	32,13°	17,63°	8,43°
	Extrêmes	20°-58°	9°-19°	16°-46°	4°-47°	0°-15°
Post-opératoire	Moyenne	16,10°	9,93°	26,27°	12,73°	7,23°
	Extrêmes	-12°-43°	2°-15°	10°-40°	0°-49°	0°-24°
Gain en correction		18,07°	5,2°	5,86°	4,9°	1,2°
« p » de Pearson		< 0,0001	< 0,0001	< 0,0001	0,002	0,28

**Tableau 2:** les complications de l’ostéotomie Scarf dans notre série

Complications		Nombre de cas		Pourcentage	
Infection		1		3,3 %	
Algodystrophie		1		3,3 %	
Retard de consolidation		1		3,3 %	
Métatarsalgies		2		13,3 %	
Troubles de correction	Hyper	2	6	6,7 %	20%
	Hypo	4		13,3 %	

## Discussion

Notre série est certes courte mais elle corrobore avec ce qui a été proposé par la plupart des travaux de la littérature sur l'ostéotomie de Scarf dans le traitement de l'hallux valgus. Le taux de patients satisfaits ou très satisfaits correspond à celui de la littérature [9-15]. A l'encontre de Groulier [16], nous n'avons pas rapporté de corrélations entre satisfaction des patients et l'âge au moment de la chirurgie ou l'importance de la déformation en préopératoire. Cependant on a constaté une corrélation significative entre satisfaction des patients et les métatarsalgies ainsi que les douleurs au niveau de l'exostose. En fait, les patients gardant des douleurs sont très déçus ce qui nous emmène à réaffirmer que la chirurgie de l'hallux valgus reste avant tout une chirurgie fonctionnelle plus qu'une chirurgie esthétique. Les résultats radiologiques sont moins importants aux yeux des malades mais néanmoins il faut retrouver une anatomie normale afin d'éviter les récidives [17]. Au dernier recul, nous avons obtenu une diminution significative des différentes déformations. Ces corrections sont presque équivalentes à celles relevées dans les différentes séries de Scarf avec fixation comme le montre le Tableau 3. L'angle métatarso-phalangien est corrigé en dépassant légèrement les limites supérieures de la normale et le valgus épiphysaire de la tête de M1 persiste malgré la correction élevée. Comme Bonnel [11], on a retrouvé une corrélation post-opératoire entre les angles métatarso-phalangien et articulaire distal métatarsien témoignant de l'interaction entre ces deux mesures. Les complications de ce type de chirurgie sont dominées par les hypo-corrections et les hyper-corrections [18]. L'hypo correction est définie dans la littérature par un angle métatarso-phalangien supérieur à 25° alors que la récidive correspond à un angle supérieur à 30° [19]. Le symposium de la SOFCOT en 2002 [20] avait conclu à un taux de récidive de 3 à 8% et il est de 3,3% dans notre série. Les causes de cette hypo-correction sont variées et représentées essentiellement par une arthrolyse latérale incomplète et/ou par la persistance d'hallomégalie en post-opératoire [18, 19, 21]. L'hallux varus iatrogène survenant après ostéotomie de Scarf est incontestablement mal vécu par le malade et le chirurgien vu qu'il est douloureux et invalidant. Cette complication relève de plusieurs facteurs: une arthrolyse latérale excessive ou une exostosectomie généreuse empiétant sur le cartilage de la tête de M1 [18, 21, 22]. Comme l'ont énoncé Groulier [16], Weil [23] et Jardé [12], nous sommes convaincus que toute chirurgie de l'hallux valgus symptomatique doit s'adresser à l'avant-pied dans son ensemble comme étant une seule structure architecturale et fonctionnelle d'où la nécessité de gestes associés. L'ostéotomie phalangienne de raccourcissement ou de varisation [24] a pour but de corriger l'hallomégalie et le valgus phalangien persistant après l'ostéotomie métatarsienne afin d'obtenir un avant-pied grec ou carré [25]. Cependant, il n'y a pas de corrélation entre les résultats mais ça reste un geste essentiel afin d'éviter les récidives comme le montre Barouk [26] qui associe cette ostéotomie dans 80% des cas. Nous pensons comme Barouk [26], Berg [27], Borelli [23], Jardé [12] et Garrido [24] que l'ostéotomie Weil est le geste associé le mieux adapté pour pallier les troubles structuraux engendrés ou aggravés par l'hallux valgus et respecter l'harmonie de la parabole métatarsienne. Cette ostéotomie faisait partie de notre protocole dans 60% des cas totalisant 24 gestes sur 18 pieds. Elle n'a pas entraîné de modifications significatives de la formule métatarsienne de l'avant-pied mais elle avait une action antalgique significative sur les métatarsalgies. Dans notre expérience, cette ostéotomie n'a pas eu d'influence sur le résultat subjectif des patients ni sur le score global de Groulier mais nous pensons, comme convenu dans la conférence de la SOFCOT de 2005 [21], qu'il est indispensable de la réaliser s'il existe une défaillance fonctionnelle symptomatique des rayons externes malgré le risque de raideur métatarso-phalangienne persistant même devant la planification d'une rééducation spécifique. Kristen [14] définit les déformations maximales corrigeables par ostéotomie de Scarf par un angle métatarso-phalangien de 40° et un métatarsus varus de 20° mais la conférence de la SOFCOT de 2005 (21) affirme que le Scarf répond à la majorité des indications y compris les déformations sévères [28], les formes juvéniles [29] et même le pied rhumatoïde [27].

**Tableau 3:** résultats de la correction des déformations angulaires

	M1 – P1		M1 – M2		DMMA	
	pré	post	pré	post	pré	post
Gayet(9)	37°	21°	15°	10°	-	-
Plaweski(10)	32,5°	18°	14,5°	10°	-	-
Seite(2)	37°	19°	15	8,7°	-	-
Crevoisier(13)	32°	17°	16°	10°	13°	10°
Salmeron(17)	28°	17°	15°	9°	13°	11°
Kristen(14)	32,5°	13,5°	14,4°	7,9°	12,2°	8,6°
Freslon(19)	31,2°	17,5°	12,1°	10,4°	13,3°	11,1°
Malviya(25)	37,9°	16,4°	18,8°	7,5°	19°	9,9°
Berg(27)	32°	18°	15°	9°	-	-
Hassoun(15)	36,3°	16,3°	15,8°	7,3°	28,5°	14,8°
Garrido(24)	34°	17,4°	15°	9,4°	18,4°	9,4°
Jardé(12)	39,8°	22,7°	15,8°	10,4°	-	-
Notre série	34,17°	16,1°	15,13°	9,93°	17,63°	12,73°

## Conclusion

L'ostéotomie de Scarf avec fixation est un geste simple, sûr, stable et efficace qui offre des résultats immédiats. Cependant, les bons résultats ne sont obtenus qu'au prix d'une évaluation préopératoire soigneuse, d'une correction peropératoire adéquate et d'une technique chirurgicale rigoureuse qui nécessite une réflexion et une planification prenant en charge globalement l'avant-pied. Cette ostéotomie garde toujours sa place pour la prise en charge de l'hallux valgus symptomatique devant les techniques mini-invasives ou percutanées.

### Etat des connaissances actuelles sur le sujet

- La chirurgie de l'hallux valgus par l'ostéotomie de Scarf a prouvé son efficacité;
- Plusieurs facteurs peuvent influencer le résultat fonctionnel de cette chirurgie.

### Contribution de notre étude à la connaissance

- Expose les résultats de note série;
- La prise en charge de l'hallux valgus doit s'intégrer dans une prise de l'avant pied dans sa globalité;
- Les gestes associés à l'ostéotomie du Scarf peuvent améliorer les résultats de cette chirurgie.

## Conflits des intérêts

Les auteurs ne déclarent aucun conflit d'intérêts.
